# Rapid Establishment of a Biospecimen Resource To Study the Global Impact of COVID-19 Vaccines

**DOI:** 10.1128/spectrum.02117-23

**Published:** 2023-06-27

**Authors:** K. E. Berliner, T. Ezzelle, T. Klenk, G. Dunn, J. Sischo, D. Campbell, K. T. McKee

**Affiliations:** a BioIntegrity, LLC, Glenwood Springs, Colorado, USA; b Allucent, Cary, North Carolina, USA; University Paris-Saclay, AP-HP Hôpital Antoine Béclère, Service de Microbiologie, Institute for Integrative Biology of the Cell (I2BC), CEA, CNRS

**Keywords:** COVID-19, biobank, biorepository, biospecimen, clinical microbiology, clinical trial, quality assurance, training, vaccines

## Abstract

The emergence and explosive spread of the severe acute respiratory syndrome coronavirus 2 (SARS-CoV-2) in 2019 highlighted the need to rapidly develop curated biobanks to inform the etiology, diagnosis, and treatment options for global outbreaks of communicable diseases. Recently, we undertook efforts to develop a repository of biospecimens from individuals aged 12 and older who were to be vaccinated against coronavirus disease 19 (COVID-19) with vaccines developed with support from the United States Government. We planned to establish 40 or more clinical study sites in at least six countries to collect biospecimens from 1,000 individuals, 75% of whom were to be SARS-CoV-2 naive at the time of enrollment. Specimens would be used to (i) ensure quality control of future diagnostic tests, (ii) understand immune responses to multiple COVID-19 vaccines, and (iii) provide reference reagents for the development of new drugs, biologics, and vaccines. Biospecimens included serum, plasma, whole blood, and nasal secretions. Large-volume collections of peripheral blood mononuclear cells (PBMCs) and defibrinated plasma were also planned for a subset of subjects. Participant sampling was planned at intervals prior to and following vaccination over a 1-year period. Here, we describe the selection of clinical sites for specimen collection and processing, standard operating procedure (SOP) development, design of a training program for tracking specimen quality, and specimen transport to a repository for interim storage. This approach allowed us to enroll our first participants within 21 weeks from the study’s initiation. Lessons learned from this experience should benefit the development of biobanks in response to future global epidemics.

**IMPORTANCE** The ability to rapidly create a biobank of high-quality specimens in response to emergent infectious diseases is critical to allow for the development of prevention and treatment, as well as to effectively monitor the spread of the disease. In this paper, we report on a novel approach to getting global clinical sites up and running within a short time frame and to monitor the quality of specimens collected to ensure their value in future research efforts. Our results have important implications for the monitoring of the quality of biospecimens collected and to design effective interventions to address shortcomings, where needed.

## OBSERVATION

Rapid development of diagnostic, treatment, and prevention measures in the setting of emergent and rapidly spreading infectious diseases is facilitated by the existence of biospecimen collections reflective of the populations targeted for receipt of these interventions. The ability to develop quality interventions quickly can significantly reduce the impact of these diseases on individuals and on their larger communities. Creation of a high-quality biobank of diverse specimen types requires a variety of resources that include relationships with appropriate collection and processing sites, understanding local regulatory and ethics requirements related to study protocol approval and patient consent, specimen shipping, subject matter expertise relevant to specimen types and sampling procedures, access to skilled individuals who will collect, handle, process, and store biomaterials, a reliable logistic infrastructure experienced in international biospecimen transport, and supplies and reagents to support these activities. Here, we describe our experience in establishing a global resource for collecting biospecimens obtained from SARS-CoV-2-vaccinated individuals. Biospecimens obtained (serum, plasma, peripheral blood mononuclear cells [PBMCs], whole blood, and nasal swabs) were to be used for testing, assay development, and quality assessment, as well as for future testing needs that may arise. While this study was directed at following individuals before and after COVID-19 vaccination, the steps taken here are broadly applicable to the rapid development of biobanks for other disease-related investigative needs.

### Biospecimens.

Specimens collected under this protocol included whole blood for subsequent isolation of PBMCs using cell preservation tubes (CPT tubes, blue top; Becton, Dickinson; catalog no. 362761), red-top serum separator tubes (SST tubes; Greiner Bio-One; catalog no. 455092) for clot activation, Pax gene tubes for DNA (Becton, Dickinson; catalog no. 761165), and Tempus blood tubes (Applied Biosystems; catalog no. 4342792) for RNA isolation. Blood-derived specimens were collected in the above order as described in guidelines developed by the Clinical and Laboratory Standards Institute (1, 2) and were performed according to the manufacturer’s recommendations.

Nasal swab samples were collected using Nasosorption FX-I devices containing synthetic absorptive matrices (SAM; Hunt Developments; catalog no. NSFL-FXI-11) to sample the mucosal lining fluid of the upper and lower airway. Briefly, the Nasosorption FX-I applicator swab was removed from a cryotransport tube, inserted into the participant’s nostril, and placed against the inferior turbinate. The participant was asked to press a finger against the side of the nostril that held the swab for 60 s. The swab was then removed, returned to the cryotransport tube from which it originated, and immediately transferred to 70°C. Elution of the SAM filters was accomplished by cutting the handle of the nasosorption device using sterile disposable forceps and transferring the SAM filter in a 2-mL microcentrifuge tube containing phosphate-buffered saline (PBS). The sample was then vortexed to wash the SAM filter clean of loosely attached fluids and biomolecules. To ensure full sample recovery, centrifugal elution was performed by adding the moist SAM to a spin filter minicolumn inserted into the same 2-mL microcentrifuge tube used for washing. Samples were centrifuged for 20 min at 16,000 × *g* at 4°C. The spin column and SAM were subsequently removed and disposed of. Finally, the eluate was aliquoted into labeled cryogenic tubes recording the total volume of eluate and the volume in each aliquot. Samples were stored upright at −80°C.

PBMCs were isolated using a protocol adapted from the Division of Microbiology and Infectious Diseases (DMID)/NIAID standard operating procedure (SOP) for PBMCs and the Plasma Processing SOP for Vaccine and Treatment Evaluation Units (VTEUs; V6.0, September 2021). In summary, whole blood was collected in CPT tubes and processed at room temperature (15 to 30°C) within a 4-h period following blood draw. Blood was centrifuged at 1,800 × *g* for 30 min without brake followed by the removal of plasma by aspiration without disturbing the mononuclear cell layer below. The cell layer (mononuclear cells and platelets) was then harvested with a pipette, transferred to a 50-mL conical centrifuge tube, washed twice with 3 volumes of phosphate-buffered saline (PBS), and centrifuged at 300 × *g* without braking. Prior to the final centrifugation, cells were counted using either automated or manual counting methods (depending on the capabilities of the processing site), and the final cell pellet was resuspended to 5 × 10^6^ cells/mL in cold (4 to 8°C) freezing medium (10% dimethyl sulfoxide [DMSO] in fetal bovine serum [FBS]). Cells were transferred to rate-controlled freezers (Corning CoolCell FTS30 or equivalent) within 10 min in freezing medium and placed at −70°C overnight. Cryopreserved cells were then transferred to liquid nitrogen vapor (LN_2_) for local storage within 24 h and transferred to the interim repository in dry LN_2_ shipping Dewars.

To facilitate the collection of high-quality specimens, we developed a Biospecimen Quality Assessment Plan (QAP) that consisted of four components: 1, site selection criteria and evaluation; 2, staff training and qualification; 3, internal quality control (IQC); and 4, external quality control (EQC). Component 1 was relevant to the collection of all specimens under this study, component 3 was uniquely developed for PBMC isolation, and component 4 applied to PBMC isolation and nasal swab processing.

### Site selection criteria and evaluation.

Criteria for site selection included sites in countries which, at the time of initial study activities, reported low COVID-19 prevalence and vaccine coverage and could provide a diverse sampling of age, sex, and race/ethnicity among potential study participants. Specific site selection considerations included the following: country-specific regulatory and startup timelines; the potential availability of adult and adolescent study participants meeting enrollment criteria; current and projected country-specific and local (as available) COVID-19 vaccination rates and disease activity; local authorization of, and access to, eligible COVID-19 vaccines; investigator and study site experience in conduct of clinical research; qualifications and experience of site laboratory personnel (and the ability of those personnel to perform work according to Good Clinical Laboratory Practices [GCLP], WHO, 2009) with particular emphasis on PBMC processing experience and expertise; and site space availability, infrastructure, and capability to meet sample processing and storage requirements.

Sites were screened by questionnaire to assess capabilities for collection, processing, cryopreservation, and transport of biospecimens. For those sites at which the laboratory processing the biospecimens would be external to that at which the specimens were collected, the proximity needed to be such that processing could be initiated within a 4-h window following collection (3, 4). Under these conditions, sites needed to have designated couriers that could transport specimens and ensure that temperatures appropriate for transport could be maintained.

Sites that met requirements for study participation were further evaluated with either in-person or virtual site evaluation visits (SEVs) by trained experts to assess facilities, equipment, existing standard operating procedures (SOPs) relevant to this study (e.g., staff training, equipment maintenance and qualification, supply records, etc.), staffing requirements, and other elements needed for the effective support of this study. Any gaps in equipment, supplies, or training were immediately addressed (e.g., new equipment purchased, LN_2_ sources identified, educational interventions provided, etc.).

Sites were required to provide documentation demonstrating prior proficiency in processing PBMCs (it was acceptable that the prior experience may have used a different procedure than what was required under the current study) as reflected in recovery and viability metrics. SEV reports were evaluated by laboratory-experienced study personnel to assess any potential shortcomings and, if found, to determine ways to address those so that the site could successfully participate. Once sites were approved for participation and before the first participants were enrolled, a site initiation visit (SIV) was performed by a trained clinical research associate (CRA) to ensure all aspects of the study were understood and to address site staff training needs as necessary.

### Staff training and qualification.

Selected sites were required to have experience collecting each of the specimen types covered by this study according to GCLP. Special emphasis was placed on prior experience in collecting and processing PBMCs. Staff participating in biospecimen collection and processing were required to follow study-specific SOPs and were trained on these procedures prior to processing samples collected from study participants. Staff processing PBMCs were required to receive additional training, as described below.

Due to the importance of PBMC specimen quality for assurance of their utility in downstream assays, special attention was given to training, assessment, and oversight of study site staff involved in collection and processing of these samples. In addition to comprehension of study-specific SOPs, a variety of educational materials were created and evaluation requirements were established, to ensure that PBMCs developed under this protocol met acceptable quality standards. PBMC processing staff were required to view a presentation on the essential steps in the procedure that was presented live and recorded by a subject matter expert (SME). Following the observance of the presentation, processing staff were required to obtain a passing score of 80% on a 15-question multiple-choice exam covering the process. If a staff member failed to pass the examination after three tries, the site Principal Investigator would be notified, and support would be provided by study personnel to identify gaps in knowledge to allow the staff member to be successful in their attempt to pass the exam. Should the staff member fail to be successful after three additional tries, they would not be allowed to participate in PBMC processing for this study. Laboratory managers were responsible for documenting training in accordance with site-specific SOPs.

Once the above steps had been completed, staff with prior PBMC processing experience received a “conditional qualification” and would be able to begin processing PBMCs obtained from study participants. Staff members became fully qualified once they completed three consecutive PBMC processing events, with results in the acceptable ranges for the designated internal quality control (IQC) indicators (see below) documented.

In addition to the training described above for individuals with prior PBMC processing experience, staff without prior PBMC processing experience were required to receive hands-on training by observing the procedure being performed by fully qualified personnel and subsequently being observed performing the procedure until the laboratory manager deemed that they could perform the procedure independently. Once this milestone had been achieved, the new trainee would be designated “conditionally qualified” and could proceed with the pathway to full qualification as described above.

Qualification for all processing staff would be maintained if the technician was actively processing specimens during the month with results documented to be in the acceptable ranges for the designated internal quality control (IQC) indicators (see below). A list of conditional and fully qualified technicians and the dates of those qualifications were maintained in the laboratory site file. Laboratory managers were to communicate with CRAs when new staff were added.

In addition to the training described above, detailed instructions were developed to instruct processing staff on how tracking forms were to be completed and results conveyed. These tracking forms not only included information about PBMC processing but also contained information about plasma hemolysis, allowing for a quality assessment of this material type as well.

### IQC for PBMCs.

Building on the work of the HIV Vaccine Trial Network (5) (HVTN) and other large clinical trial networks (6) collecting and processing PBMCs, we followed five key internal quality control (IQC) indicators to monitor initial PBMC quality: (i) fresh cell yield per milliliter of whole blood, (ii) time from blood collection to the start of processing, (iii) total processing time (the time at which processing of blood samples began and cryopreservation was initiated), (iv) the total time to cryopreservation (the time at which the specimens were collected through the initiation of cryopreservation), and (v) initial PBMC viability. These data were collected by study site personnel, recorded on procedure-specific forms, and entered into a study-specific tracking form. Completed worksheets were entered by lab staff into a secure web portal for review by the Study Sponsor.

Data entered on tracking forms were reviewed by the site after each processing event to ensure that each IQC indicator was within acceptable ranges (Table 1). Additionally, sites completed a spreadsheet entitled “PBMC Processing Results” using data collected on tracking forms. These spreadsheets were submitted for review by a project SME on a weekly basis, and a detailed assessment was created for review and response by the site Principal Investigator. If needed, a site visit by an SME would be planned to review all aspects of the processing efforts. While every attempt was made to ensure that IQC results were within acceptable ranges, it was recognized that some technical personnel and/or study sites would be unable to achieve this requirement. In this event, they would be excluded from further work in the study. Data uploaded into a SharePoint site would allow for overall site review as well as review by technicians.

**TABLE 1:** Internal quality control ranges for PBMC isolation

Parameter	Below range	Acceptable range	Above range
Fresh cell yield/mL of whole blood	<0.8 × 10^6^	0.8 × 10^6^–3.2 × 10^6^	>3.2 × 10^6^
Time from blood collection to the start of processing		≤240 min	
Total processing time	<70 min	70–240 min	>240 min
Total time to freeze	<85 min	85–480 min	>480 min
Initial PBMC viability		≥95%	

### EQC for PBMCs and for nasal swab samples.

**(i) PBMCs.** To determine PBMC quality once cells had reached the repository at which they would be ultimately stored, external quality control (EQC) measures including a postthaw cell count, viability assessment, and recovery (comparing the number of cells that were alive with the number of live cells that were reportedly put into the sample) were planned. Subsequent analyses would involve a secondary measurement of cell count following an overnight incubation at 37°C with 5% CO_2_ as described for the HVTN (5) and for the Center for HIV/AIDS Vaccine Immunology (CHAVI) consortium (6). As with IQC results, EQC findings were to be shared with the sites as soon as they were available, and every attempt was made (e.g., appropriate interventions by an SME) to ensure that results could be brought into acceptable ranges. Should these attempts fail, the site would be excluded from future work on the study.

**(ii) Nasal swab samples.** Nasal swab secretions collected for mucosal antibody analyses and other assessments were eluted from Nasosorption FX-I devices as described by the manufacturer (Hunt Developments, Ltd.). Quality control of eluates obtained from Nasosorption FX-I devices was performed using a commercially available Pierce Rapid Gold bicinchoninic acid (BCA) protein assay kit (ThermoFisher; catalog no. A53227) to quantitate total protein in the eluates. The rationale for selection of this generic method of quantification was based on the yet-to-be-determined/unspecified downstream applications in which samples would ultimately be employed. The presence of protein in the nasal eluate samples was therefore used as a surrogate for adequacy of specimen collection and processing. The BCA protein assay has a linear range of 20 to 2,000 μg/mL, and the laboratory established limits of detection (blank plus 3 standard deviations) as 2 μg/mL total protein using the microplate procedure. Consequently, 2 μg/mL of total protein was selected for our lower limit of detection (Table 2).

**TABLE 2:** Acceptance criteria for extracted nasal swab specimens

Protein concn acceptance criterion	QC pass/fail	Reporting status
≥2 μg/mL	Pass	Detectable
<2 μg/mL	Fail	Undetectable

### Conclusions.

The rapid assembly of staffing, appropriate laboratory settings, standard operating procedures (SOPs), and tracking systems to support the development of a biospecimen resource in response to an emerging global health epidemic requires reliance on a diverse array of factors. Within 21 weeks from the initiation of this project, four study sites (three in South Africa and one in the United States) had been identified, evaluated, qualified, and approved for participation. Thirty-three participants were enrolled, consented, and contributed biospecimens.

While the foundation for this effort was comprehensive, the timing at which the effort was initiated prevented the completion of the full aims of the project. By the end of 2021 COVID-19 vaccines had become increasingly accessible in our target countries. At the same time, rapid and unimpeded spread of the Delta and Omicron SARS-CoV-2 variants frustrated efforts to identify COVID-19-naive individuals to participate in the study. In addition, supply chain shortages affected the ability to get all collection kit supplies to sites in a timely manner. Based on these combined events, in early March of 2022, a decision was made to suspend the study.

Despite our inability to fully execute this project as initially planned, the framework developed here provides for a timely assessment of specimen quality, an essential component for the development of all specimen resources. For PBMCs, there are numerous activities that follow cell count and viability assessments that affect quality and, ultimately, functionality and utility. Examples include delayed exposure to DMSO prior to freezing, poor cold-chain management of specimens during handling, temperature excursions during shipment, etc. (7). Through staff training assessments and quantitative tracking systems, we could immediately identify any specimens that fell outside acceptable ranges set for processing times, yield, and viability. As noted by the HVTN (5), “an initial competency assessment was not sufficient to monitor ongoing PBMC quality in a manner that reflected the performance of tasks when a laboratory is working under the challenges of an actual clinical trial”; we therefore established an external PBMC quality assessment program to ensure that cells collected and stored would serve their expected purposes when used.

Likewise, EQC measures developed for nasal swab extractions would allow for a rapid assessment of mucosal secretion quality for future immunological testing. Taken together with the PBMC quality assessments, these steps would provide for a timely determination of additional training needs for staff and the implantation of appropriate interventions as well as an approach to predicting the future value of the specimens collected.

We add here a note that when considering the development of a quality management system for the establishment of a new biobank, it must be emphasized that the quality parameters specific to products used in our study (e.g., CPT tubes, SST tubes, etc.) should not be extrapolated to work for similar products from other vendors. Each collection effort undertaken should include some method to ensure that the techniques and procedures used under relevant SOPs conform to the product-specific guidelines and that the specimens collected meet preestablished quality standards.

Finally, the ability to coalesce the essential elements for quality specimen collection—site selection, development of SOPs, staff training, and monitoring of biospecimen quality—is relevant to the development of all biospecimen resources. The steps taken for this study, while suspended prior to the full accomplishment of initial study goals, should be relevant to future efforts at establishing important biospecimen resources for future research.
